# Supplementary material for: Copy Number Variations of KLF6 Modulate Gene Transcription and Growth Traits in Chinese Datong Yak (Bos Grunniens)
Source: Animals (Basel). 2018 Aug 21;8(9):145. doi: 10.3390/ani8090145 (PMC6162419; doi:10.3390/ani8090145)
Supplement: Supplementary file 1 [file animals-08-00145-s001.zip › Ethinic statement.pdf]

Legal Permission Animals Use for Research

The study was conducted between September 2016 and December 2017 by Prof. Ping Yan and Dr.Habtamu Abera Goshu at Key Laboratory of Yak Breeding Engineering of Gansu Province, Lanzhou Institute of Husbandry and Pharmaceutical Sciences, the data obtained from yak breeding cooperatives of Gansu and Qinghai Province, China. All blood sample collection and body measurements were strict accordance of the guide for the Care and Use of Laboratory, Animals, Lanzhou Institute of Husbandry Animal and Pharmaceutical Sciences, China. Also, all animals were slaughtered under anesthesia, and all necessary efforts were made to minimize risk of suffering. Thus, we agree to perform the research on the yak and the legal certificate number was SCXK (Gan) 2014-0002.

中国农业科学院兰州畜牧与兽药研究所

Lanzhou Institute of Husbandry and Pharmaceutical Sciences
Chinese Academy of Agricultural Science

26/03/2018
