# Supplementary material for: Copy Number Variations of KLF6 Modulate Gene Transcription and Growth Traits in Chinese Datong Yak (Bos Grunniens)
Source: Animals (Basel). 2018 Aug 21;8(9):145. doi: 10.3390/ani8090145 (PMC6162419; doi:10.3390/ani8090145)
Supplement: Supplementary file 1 [file animals-08-00145-s001.zip › supplementary.pdf]

Table S 1. Distribution of the *KLF6* gene CNV types among breeds.

Breeds	Sample size	NV type		
		Gain	Loss	Normal
Datong yak	30	6.7% (2)	26.7% (8)	66.7% (20)
Polled yak	30	96.7% (29)	-	3.3% (1)
Tianzhu Yak	30	10.0% (3)	56.7% (17)	33.3% (10)
Plateau Yak	30	6.7% (2)	56.7% (17)	36.7% (11)
Gannan yak	30	10.0% (3)	60.0% (18)	30% (9)
Overall	150	26% (39)	40% (60)	34% (51)

Table S2. Pairwise comparison of CNV of the *KLF6* gene in five Chinese domestic yak breeds.

Yak breeds	Mean Diff.	*p*-Value	q or t ratio	Significance	95% CI of diff
Datong vs Polled	−1.3	0.0001	12	***	−1.7 to −0.83
Datong vs Tianzhu	0.14	0.35	1.2	NS	−0.29 to 0.57
Datong vs Gannan	0.45	0. 012	4.1	*	0.020 to 0.88
Datong vs Plateau	0.24	0.1	2.2	Ns	−0.19 to 0.67
Polled vs Tianzhu	1.4	0.0001	13	***	0.97 to 1.8
Polled vs Gannan	1.7	0.0001	16	***	1.3 to 2.1
Polled vs Plateau	1.5	0.0001	14	***	1.1 to 1.9
Tianzhu vs Gannan	0.31	0.085	2.9	NS	−0.12 to 0.74
Tianzhu vs Plateau	0.11	0.57	0.97	NS	−0.32 to 0.54
Gannan vs Plateau	−0.21	0.20	1.9	NS	−0.64 to 0.22

*** The variance significant at $p < 0.0001$, and * $p < 0.05$, NS, Non significant
